# Vitamin D and LC-PUFA and the Presence of Fetal Heart Defects—A Further Part of a Case-Control Study

**DOI:** 10.3390/nu17010018

**Published:** 2024-12-24

**Authors:** Agnieszka Kolmaga, Elżbieta Trafalska, Ewelina Gaszyńska, Anna Gawron-Skarbek, Sławomir Witkowski, Julia Murlewska, Maria Respondek-Liberska, Iwona Strzelecka

**Affiliations:** 1 Department of Nutrition and Epidemiology, Medical University of Lodz, 90-752 Lodz, Poland; 2 Department of Geriatrics, Medical University of Lodz, 92-209 Lodz, Poland; anna.gawron@umed.lodz.pl; 3 Medical Faculty, Ludwik Rydygier Collegium Medicum Bydgoszcz, 85-067 Bydgoszcz, Poland; 4 Department of Prenatal Cardiology, Polish Mother’s Memorial Hospital Research Institute in Lodz, 93-338 Lodz, Poland; 5 Department of Diagnoses and Prevention of Fetal Malformations, Medical University of Lodz, 90-419 Lodz, Poland

**Keywords:** pregnancy, congenital heart defects, vitamin D, LC-PUFA, DHA, supplementation

## Abstract

Introduction: The relationship between diet of mothers, including supplementation of vitamin D and Long Chain Polyunsaturated Fatty Acids (LC-PUFA), and the prevalence of congenital heart defects (CHD) in the fetus has not been sufficiently studied. The aim of the study was to investigate the relationship between the intake of vitamin D and LC-PUFA by mother (from diet and with supplementation, including its time of implementation and applied dose), and the risk of CHD in the fetus. Methods: This was a case—control study with the participation of a total of 79 women with prenatally diagnosed CHD in the fetus and 121 women without CHD in the fetus. The Food Frequency Questionnaire (FFQ) was used with particular emphasis on vitamin D and DHA supplementation. A univariate logistic regression model was used to evaluate the relationship between selected dietary data and the risk of CHD in the fetus. Results: In the group of females without CHD in the fetus, the mean values of vitamin D intake, including supplementation, and dietary LC-PUFA + DHA from supplementation, were higher than in women with CHD in the fetus (41.3 ± 24.7 vs. 34.7 ± 27.8 μg; *p* = 0.02 and 831.0 ± 280.1 vs. 767.7 ± 287.6 mg; *p* = 0.008, for vitamin D and LC-PUFA + DHA respectively). No significant relationship was found between vitamin D intake (including supplementation) or between LC-PUFA + DHA supplementation by pregnant women, and the presence of CHD in their children. Conclusions: It seems that increased maternal intake of vitamin D and LC-PUFA, including DHA, through supplementation, may protect fetus against CHD, but the relationship between the occurrence of CHD and diet in this area need further studies.

## 1. Introduction

It is confirmed that congenital heart defects (CHD) are the most common congenital defects that affect 1 in 100 newborns worldwide [1]. They cause significant social, medical and financial burdens on society and the family [1,2,3]. Therefore, primary prevention is so important due to the fact that it would significantly reduce these burdens on cardiological patients. This requires a better understanding of the factors that are involved in the development of CHD [2].

Some deficiencies of nutrients may be associated with numerous developmental defects or fetal anomalies [4]. Adequate nutritional status of a woman, even before conception, and appropriate nutritional customs in pregnancy are essential to prevent numerous maternal and fetal pathologies [5,6]. The critical time for intervention in terms of preventing birth defects may be early pregnancy, when the fetal organ systems begin to develop [3]. Given the critical period of heart development, which is 3–8 weeks, the most important is periconceptional nutrition [2].

Vitamins, minerals, and omega-3 fatty acids play an important role during this period because they ensure proper fetal development and prevent many pregnancy complications or fetal birth defects [7]. Therefore, it is essential to ensure the proper status of vitamins and other nutrients in pregnant women, especially since some deficiencies are all too common among women who plan a pregnancy or pregnant women, including deficiencies of vitamin D, folic acid, iron or iodine [7,8] or omega-3 acids [6,9].

Vitamin D is a key component in the heart development. Many cells during organogenesis contain specific VDR receptors, through which vitamin D takes an active part in the initial stages of development, mainly it can stimulate the development of the brain, liver, heart, kidneys and intestines. Vitamin D deficiency during pregnancy therefore results in disorders in organogenesis and may affect the health of the child [10,11]. It has been found that vitamin D physiologically increases its concentration by 100–200% in early pregnancy [11].

The use of supplements during pregnancy is common practice and can help cover the increased demand. In addition to vitamin D, essential unsaturated fatty acids are crucial for the proper development of the fetus [4]. In addition, during pregnancy a decrease in the level of DHA in the mother’s serum and the potential reduction of the maternal reserves are observed. As a result, pregnancy is a period of an increase in the demand for DHA by 100–200 mg/day. Only 2% of pregnant women adhere to a diet that fulfills the recommended DHA intake [12]. Therefore, it is recommended to begin supplementing omega-3 fatty acids before 20 weeks of pregnancy. They have a beneficial effect on the mother (e.g., reduced symptoms of postpartum depression, reduced cardiovascular risk) and the fetus, which is associated with an anti-inflammatory role [4,13]. Omega-3 fatty acid supplementation may reduce oxidative stress. However, this relationship is rarely studied during pregnancy [14] and similarly the *n*-3 intake in the context of congenital heart defects. Previous studies showed that low vitamin D status/low dietary intake in pregnant women was associated with the birth of a child with a congenital heart defect [11,15]. It was suggested that future studies should focus on the benefits of a diet rich in vitamin D and/or supplementation in the period preceding pregnancy [15].

This study focuses on the dietary intake and supplementation of vitamin D among pregnant women during the periconceptional period and in the first weeks of pregnancy as well as examines the association with fetal CHD. In addition, the intake and supplementation of DHA acids were assessed, as there are no studies analyzing the relationship with the occurrence of fetal heart defects. This study is a continuation of an earlier study/project in which the main goal was to assess the risk and protective factors associated with congenital heart defects in the population of pregnant women in Poland [16]. The study was carried out at the Department of Prenatal Cardiology of the Polish Mother’s Memorial Hospital in Łódź and the Department of Diagnostics and Prevention of Congenital Defects of the Medical University of Łódź in cooperation with the employees of the Department of Nutrition and Epidemiology of the Medical University of Łódź.

### 1.1. What’s New?

- This study provides new data on the intake and supplementation of vitamin D as well as long-chain fatty acids in the context of the occurrence of fetal heart defects, as there are few studies analyzing both the intake and supplementation of these ingredients before and during pregnancy in the context of congenital heart defects.
- Compared to the study group with fetal heart defects, women from the control group featured a significantly higher average intake of vitamin D and omega-3 acids together with additional supplementation. Additional supplementation of these ingredients in the diet may prove helpful and reduce the risk of occurrence of CHD in the children, which was not demonstrated in this study, however, further studies on a larger population group taking into account these ingredients would undoubtedly be needed.

### 1.2. What Are the Clinical Implications?

- It is worth indicating preconceptional supplementation of vitamin D and DHA in the recommendations for women preparing for conception and during pregnancy in order to protect their fetuses against CHD. Such supplementation may prove to be a kind of a protection against the occurrence of heart defects in children, however, insufficient intake of these important ingredients is observed.
- It is also worth educating women of reproductive age and healthcare workers to indicate and promote the necessary information on the benefits of a well-balanced diet and/or additional supplementation.
- In order to prevent congenital heart defects in the fetus, the participation of a dietician in preconception counseling, including medical, gynecological, family doctor’s offices should be considered for providing health education for future parents. Training and online counseling should be delivered for public health specialists and for those who want to have a child by providing reliable and up-to-date information on nutritional recommendations

### 1.3. The Aim of the Study

This study aimed to evaluate and analyze the intake of vitamin D and long-chain polyunsaturated fatty acids (LC-PUFA) from diet and dietary supplementation as well as their relationship in patients with congenital heart defects detected in the prenatal life (group A) and in pregnant women with a normal course of pregnancy, including normal anatomy and function of the heart (group B).

## 2. Material and Methods

### 2.1. Study Group

The study involved pregnant women with prenatally diagnosed heart defects in the single fetuses (n = 79) and those with normal pregnancies without detected heart defects and other developmental anomalies (n = 121). Participants were in their second or third trimester of pregnancy, ranging in age from 19 to 44 years.

A detailed description of the patients who participated in the study, along with the methodology, is described in the first part of the study by Kolmaga et al. entitled ‘Folic acid and selected risk factors for fetal heart defects—preliminary results of the study’ [16].

The assessment of the women’s diet before and during pregnancy was performed using a validated and modified Food Frequency Questionnaire (FFQ). The interviews allowed to gather information on the use of vitamin and mineral supplements before and during pregnancy. The participants provided detailed answers regarding the dose, frequency and duration of the use of individual or combined supplements containing vitamin D and DHA. The collected data were analyzed with the use of Dieta 6.0 software, which relies on a database of the food product composition and nutritional values developed by the NIZP-PZH-PIB staff. Based on the software, the applied methodology accounted for unavoidable losses [17]. The data regarding the intake of vitamin D and long-chain fatty acids, categorized by pregnancy trimester, were compared to up-to-date nutritional standards at the adequate intake (AI) [18].

The use of vitamin D and DHA supplementation was assessed separately and collectively with the diet. Dietary supplements available in Poland contain the discussed ingredients in different amounts depending on the time of supplementation and health needs. Therefore, a detailed interview was collected regarding the composition of the dietary supplement, the dose used and the time of starting supplementation.

### 2.2. Statistical Analysis

Numerical variables, mostly dietary data, were compared between the group with CHD in the fetus and group of women with normal heart anatomy (NHA) in their offspring with Mann-Whitney test and were presented as mean ± standard deviation. A univariate logistic regression model was used to evaluate the relationship between selected dietary data and the risk of CHD in the fetus. Detailed statistical analysis description is presented in our previous study [16].

## 3. Results

The average intake of vitamin D from the diet in both study groups, A and B, was insufficient (4.1 ± 2.5 µg and 4.4 ± 2.5 µg, respectively) according to the nutritional standards (15 µg each day). Women who used supplementation provided an average of 42.2 µg of vitamin D in group A and 43.0 µg in the control group (B) in comparison to the recommendation of 50 µg. A significant difference was found in the case of total vitamin D intake with supplementation (*p* < 0.05). A significantly lower mean was found in group A than in group B, and the corresponding means were: 34.7 ± 27.8 µg vs. 41.3 ± 24.7 µg.

**Figure 1:**
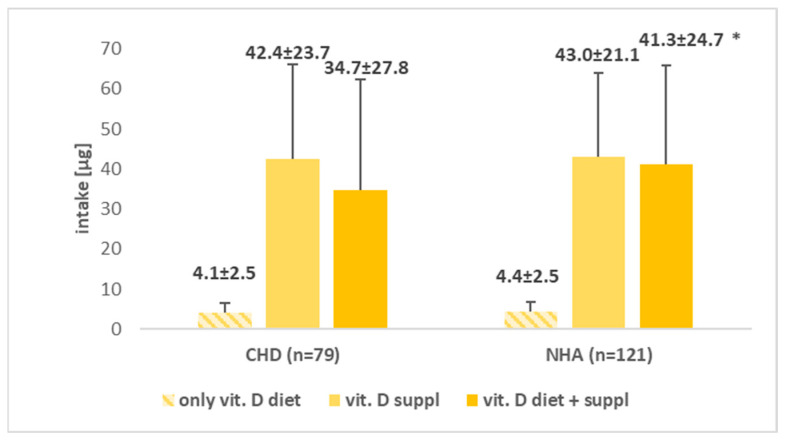
Comparison of the mean values of vitamin D variables in the group of women with fetuses diagnosed with congenital heart defects (A) and in the group of women without heart defects in fetuses (B). CHD—congenital heart defects, NHA—normal heart anatomy, *—*p* < 0.05.

**Figure 2:**
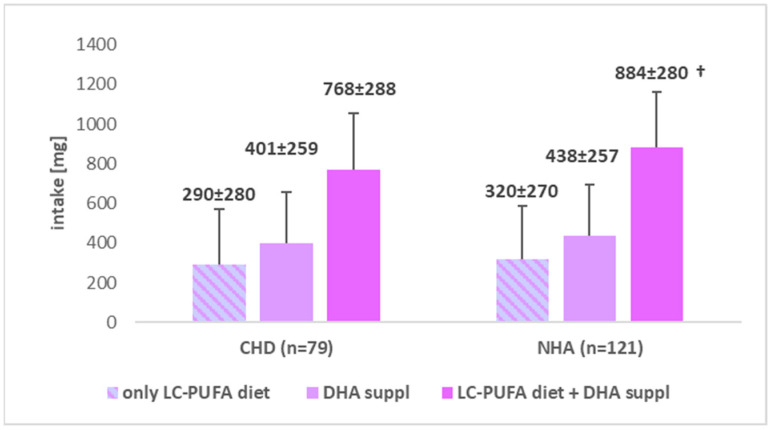
Comparison of the mean values of LC-PUFA variables in the group of women with fetuses diagnosed with congenital heart defects (A) and in the group of women without heart defects in fetuses (B). CHD—congenital heart defects, DHA—Docosahexaenoic Acid, LC-PUFA—Long Chain Polyunsaturated Fatty Acids, NHA—normal heart anatomy, †—*p* < 0.01.

The average dietary intake of LC-PUFA was also higher but not significantly (*p* > 0.05) in the control group (0.32 ± 0.27 µg) compared to the study group (0.29 ± 0.28 µg). In contrast, DHA supplementation provided an average of 401.0 ± 259.0 mg DHA in the group of pregnant women with CHD in the fetus and 438.3 ± 256.6 mg in the control group. A statistically significant difference in LC-PUFA + DHA with supplementation was observed (*p* < 0.01). The mean value was significantly lower in group A compared to group B, and the corresponding means were: 767.7 ± 287.6 mg vs. 883.9 ± 280.1 mg. The data are summarized in Figure 1 and Figure 2.

There was no statistically significant effect of the intake of vitamin D in relation to the dietary norm (*p* > 0.05). Almost all of the examined women from each group had the intake below the norm, 98.7% and 99.2%, respectively.

**Figure 3:**
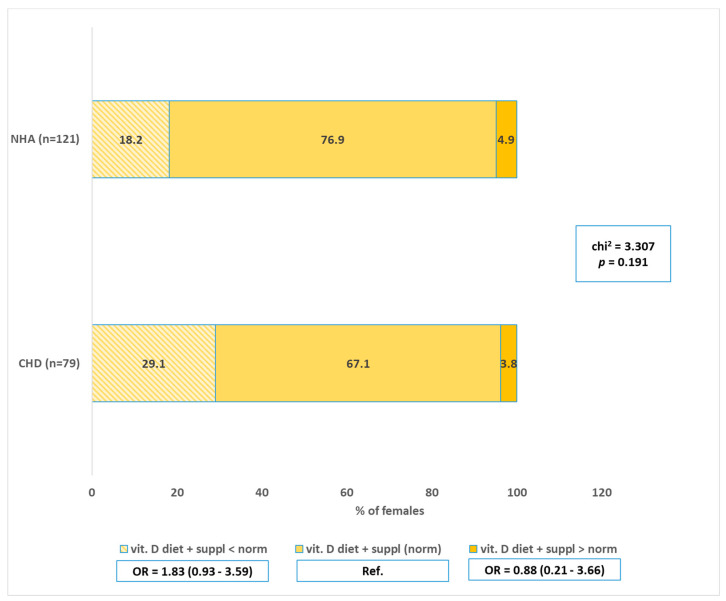
Implementation of dietary vitamin D, including supplementation in the group with CHD in the fetus and in the control group. CHD—congenital heart defects, NHA—normal heart anatomy.

Vitamin D supplements were used by 84.94% of women in general (18.27% before pregnancy and 64.51% during pregnancy) (Figure 3). Vitamin D supplementation was implemented before pregnancy by only 18.4% of pregnant women from group A and slightly more by 20.7% of pregnant women from the control group. However, it must be said that 25% of pregnant women with a fetal heart defect and 12.9% of pregnant women from the control group did not supplement vitamin D. The connection between the presence of a heart defect in children and vitamin D supplementation was also statistically insignificant (*p* > 0.05). However, it is important to highlight that the lack of vitamin D supplementation compared to supplementation before pregnancy increases the risk of the defect more than twice, although this is not statistically significant (OR = 2.17; *p* > 0.05).

LC-PUFA intake was implemented in accordance with the norm by only 12.74% of the examined pregnant women. It is worth noting that as many as 49.37% of women in group A and 42.15% of women in the control group did not implement the recommendations (below the norm of intake). No statistically significant effect of dietary LC-PUFA intake was found (*p* > 0.05).

**Figure 4:**
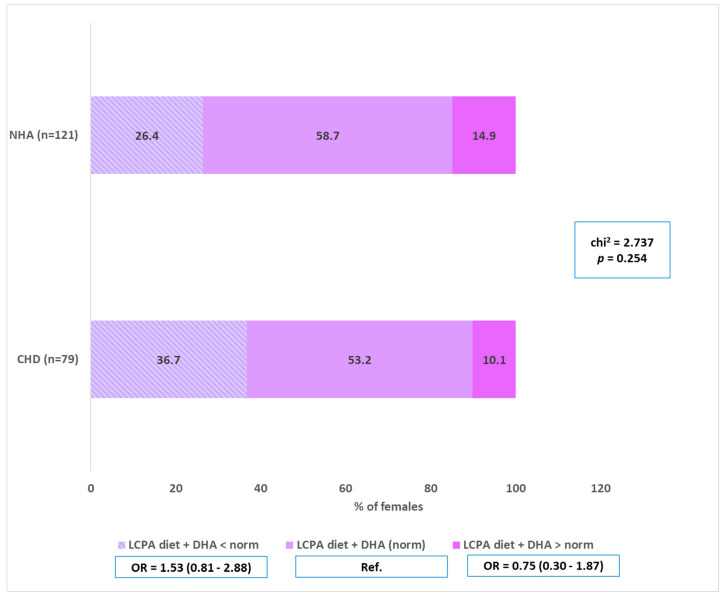
Implementation of dietary LC-PUFA, including DHA supplementation in the group with CHD in the fetus and in the control group. CHD—congenital heart defects, DHA—Docosahexaenoic Acid, LC-PUFA—Long Chain Polyunsaturated Fatty Acids, NHA—normal heart anatomy.

DHA supplementation was implemented by 66.15% of all the examined women, including 5.12% before pregnancy and 61.02% during pregnancy. Only 6.5% of pregnant women from the study group (A) took dietary supplements containing DHA before getting pregnant, whereas slightly fewer, i.e., 4.2% of women from the control group (B). No supplementation was declared by as many as 36.4% of women from the group diagnosed with a fetal heart defect and 32.2% from the group of pregnant women without a fetal heart defect. Additionally, no statistically significant impact of DHA supplementation on the incidence of heart defects in children was observed (*p* > 0.05) (Figure 4). Detailed results are summarized in Table 1 and Table 2.

## 4. Discussion

A properly balanced diet is very important in the periconceptional period to ensure a woman’s good nutritional status from the first weeks of pregnancy [19] and further efforts to balance the prenatal diet are very important [18,20]. Research indicates that women often follow an inadequate diet during this period. To address these deficiencies, the Polish Society of Gynecologists and Obstetricians recommends supplementing the five most commonly lacking nutrients, specifically folic acid, iron, iodine, vitamin D and DHA fatty acids [21]. Supplementation with vitamins and microelements during pregnancy is also recommended by other societies [2]. It has been shown that insufficient intake of essential macro- and microelements may have a negative impact on the course of pregnancy and the health of newborns. In particular, the periconceptional period is considered a critical period for the development and health of the fetus. This is due to the fact that this is the time when pregnancy-related disorders and various developmental defects occur, including congenital defects [2,15,22].

In this study, the intake and supplementation of other nutrients important for the health of women and the fetus were analyzed. These included vitamin D and LC-PUFA acids (with DHA acids) in the aspect of the presence of congenital heart defects in the studied group. No significant association was identified between the occurrence of incidence of CHD in the fetus and the intake or supplementation of vitamin D and long-chain fatty acids. However, it was observed that in the group of women without congenital heart defects in the fetus, the women had a significantly higher average intake of the analyzed ingredients by using vitamin D and DHA supplementation additionally with the diet.

### 4.1. Vitamin D—Intake and Supplementation vs. CHD

Vitamin D is a crucial vitamin for the proper development of the fetus, including maintaining bone homeostasis. Additionally, it affects the immune, hormonal and cardiovascular systems [8,19]. It is thought that during pregnancy, additional intake of vitamin D may be needed to provide protection against pregnancy complications. However, it should be noted that benefits may be observed if supplementation is started early in pregnancy, as evidence suggests that early pregnancy with adequate vitamin D nutritional status is an important determinant of maternal and neonatal health outcomes [23,24].

Vitamin D status in the body depends on dietary sources (e.g., frequent consumption of oily fish) but dietary vitamin D intake usually reaches only about 2 to 4 μg per day. Only oily fish and eggs are good sources of vitamin D, while milk and dairy products are poor sources. Vitamin D (a plant source) is also supplied by fungi [18]. Vitamin D is also synthesized in the skin under the influence of sunlight, depending on the season, and is delivered with supplementation [25,26]. Women who plan a pregnancy should take the same dose of vitamin D as adults in the overall population, preferably with monitoring of blood 25(OH)D levels. The optimal concentration of 25(OH)D during pregnancy is >30–50 ng/mL (75 nmol/L). If it is impossible to assess blood 25(OH)D levels, cholecalciferol should be supplemented at a dose of 2000 IU/day (50 μg/day) throughout pregnancy [24]. The upper limit is 4000 IU/day [6,18]. Supplementation at doses of 2000–4000 IU/day led to higher blood vitamin D levels compared to 400 IU/day. Elevated vitamin D levels were significantly associated with a reduced risk of infection, hypertensive disorders in pregnancy, and other health problems [6]. Vitamin D supplements were used by 84.94% of women in this study (only 18.27% before pregnancy and 64.51% during pregnancy). Vitamin D deficiency is a serious public health problem, the incidence of which is reported in 40–80% of the population of pregnant women. Women are provided only about 26% of vitamin D from their diet [2,6]. Low vitamin D intake is confirmed by numerous population studies [26,27], and the addition of a vitamin D supplement in some cases may not always remedy the deficiencies in the diet [26]. As suggested by Wierzejska et al., there is a need to enrich a wide range of food products because this seems to be the best way to increase vitamin D intake [26].

In Poland, a low percentage of women of reproductive age or during pregnancy who supplement vitamin D, ranging from 15.7–39% to 89% of patients, is found [8,26,28]. Similarly, in Western diets, insufficient vitamin D supplementation is observed [14]—from 33% before pregnancy to 43–71.7% during pregnancy [29,30].

By reviewing the available literature, it can be found that few studies have been conducted to analyze the association between maternal vitamin D levels/vitamin D intake and/or supplementation and the risk of developing heart defects in newborns [11,15,31].

Low maternal vitamin D levels (<50 nmol/L) in the studies of Koster et al. were associated with an approximately two-fold increased incidence of CHD in children [15]. However, this study was associated with the analysis of peripheral blood after childbirth and analysis of vitamin D status in mothers. Comparing the findings of the aforementioned study with our study is challenging because our research focuses specifically on dietary intake among pregnant women.

The authors of the study [15] concluded that it was recommended to improve the periconceptional vitamin D status of the mother because most women and girls of reproductive age have reduced vitamin D levels, and reduced vitamin D levels in the mother were associated with the birth of a child with a congenital heart defect. The first weeks of pregnancy are crucial for the development of the heart and pregnancy is often not yet confirmed. This indicates the need for an appropriate vitamin D status in the mother already in the preconception period [15].

Several other studies have examined the relationship between a specific polymorphism of the VDR gene and the risk of developing heart defects. Vitamin D status in mothers and children shortly after birth was also examined and found that vitamin D deficiency was significantly associated with an increased risk of CHD in the children [11].

In our study, in the first part of the work [16] we also assessed the occurrence of diseases in the respondents (these were mainly arterial hypertension, type 2 diabetes, thyroid diseases) and we did not find a relationship between a disease in the mother and the occurrence of CHD in the fetus. In our study, the women did not suffer from rubella, so we could not assess the relationship between the disease and vitamin D supplementation. We also did not find sufficient other studies in this field.

### 4.2. n-3 LC-PUFA (Including DHA)—Intake and Supplementation vs. CHD

A sufficient intake of omega-3 polyunsaturated fatty acids is also very important during the period of pregnancy planning and the pregnancy itself. Pregnancy is a time of special demand for these acids, especially DHA and eicosapentaenoic acid (EPA), as they play a key role in fetal growth and development, and increased intake is associated with improved maternal-fetal outcomes [8,32,33,34]. It is worth remembering that these acids have antioxidant and anti-inflammatory properties that can reduce the risk of preeclampsia or other complications [27,35].

Essential unsaturated fatty acids (EFAs) are vital for every cell in the body, serving as critical structural and functional components of cell membrane. They influence cell signaling, regulate gene expression and modulate inflammation. These are biologically active compounds that facilitate cell division and proliferation. They are crucial for embryonic and immune growth [36]. Sley et al. found in their study that supplementation with *n*-3 acids during pregnancy was associated with lower concentrations of biomarkers of maternal oxidative stress [14].

Impaired embryonic development is associated with an increased inflammatory burden, and unfavorable conditions during pregnancy can lead to fetal malformations as well as abnormal birth weight and premature birth [36].

Therefore, the demand for omega-3 acids increases significantly during pregnancy. The amount of EPA and DHA in the Polish Dietary Standards was established for pregnant women at 250 mg/d + 100–200 mg DHA/day [18]. Rich sources of DHA and EPA in the diet include fatty marine fish (salmon, herring, cod, pollock, sole), marine algae (especially from the Schizochytrium genus), seafood and krill oil [18,37]. The suggested daily intake of *n*-3 LC-PUFA during pregnancy can be met by consuming two servings of fish per week, including one serving of oily fish [18,25].

According to Roke et al., young people at an age between 18–25 years (83%) are aware that EPA and DHA are associated with the health of the heart and brain but are not aware of the recommendations regarding omega-3 fatty acids and oily fish consumption. Regular intake of omega-3 fatty acids and oily fish is generally lower than recommended [38].

It is worth mentioning that the Environmental Protection Agency (EPA) and the Food and Drug Administration (FDA) recommend that pregnant women should avoid fish species that contain high levels of mercury (e.g., king mackerel, swordfish, shark) and prefer fish and seafood with lower mercury or other contaminants (e.g., salmon, cod, catfish, shrimp, oysters) [12,18].

However, in many countries, pregnant women’s diet does not align with these recommendations [19,25,32]. Therefore, it is suggested that pregnant women who do not regularly eat fish or seafood take a supplement providing at least 200 mg DHA/day [25] or 0.5–0.6 g DHA per day in their diet. In complicated pregnancies and pregnancies at risk of premature birth, DHA supplementation should be even higher—1 g DHA per day [6,21].

As emphasized by Wierzejska et al. [39], the diet of pregnant women is largely poor in terms of DHA intake. Crucially, it is not feasible to meet the current recommendations without dietary supplementation [39]. Others are of a similar opinion—if the consumption of fish and seafood is not sufficient, the highest quality dietary supplements can provide the required amount of LC-PUFA without the risk of contamination with mercury or dioxins [32].

In our study, DHA supplementation was implemented by 66.15% of all the women examined, including 5.12% before pregnancy and 61.02% during pregnancy. However, a small percentage of pregnant women supplementing DHA is observed both in the Polish population and in other countries. Epidemiological evidence suggests that 85% of women of reproductive age have DHA deficiencies. Such deficiencies encourage the recommendation of DHA supplementation before pregnancy [12]. In the studies by Grzelak et al. [8], Grot et al. [34], Wierzejska [39] only 27.2–40% of pregnant women or women who planned pregnancy used DHA preparations despite low fish consumption. The results of Nordgren et al. [33] show that the intake of omega-3 fatty acids is also a problem in women of reproductive age and pregnant women in the United States—only 1.8% of non-pregnant women and 9.0% of pregnant women used supplements containing EPA and/or DHA, whereas in other studies *n*-3 supplements were used by <5% of women in early pregnancy [29]. In a recent study, it was reported that DHA during pregnancy (200 mg/day) was supplemented by 46.0% of the study participants, while 54.0% of women did not use such supplementation at all [12].

Strategies aimed at increasing omega-3 fatty acid intake in these at-risk groups could enhance maternal and child health outcomes [32,33]. This is especially true since pregnant women are unable to meet their omega-3 requirements from a diet containing *n*-3-rich vegetable oils or fish alone. Two servings of fish supply only approximately 100–250 mg/day of *n*-3, including 50–100 mg of DHA, therefore the remaining amount can be obtained with dietary supplements [12].

It is also worth noting that fish/seafood, in addition to being an important source of DHA and EPA, provide other essential nutrients (e.g., vitamin D, iodine, protein, selenium), which play an important role in the development of the neurological, immune and cardiovascular systems [40] and are considered beneficial for the growth and general development of the fetus [12].

In our study, women from the control group had significantly better average intake of vitamin D and omega-3 acids, which mainly resulted from the consumption of fish and seafood supported by additional supplementation compared to the intake in the study group with heart defects in the fetuses.

In one of the studies it was observed that a diet of pregnant women characterized by a high consumption of fish and seafood was associated with a reduced risk of CHD in the fetuses. The authors of the study indicate the need for further randomized interventional studies [41].

Therefore, attention should be paid to the quality of the diet and the preferential choice of food rich in critical nutrients, including vitamins, minerals and LC-PUFA, and women of reproductive age and pregnant women should be encouraged to consume specific food categories to protect against CHD in the fetus [25,42,43].

A high-quality diet rich in fruits, vegetables, legumes, nuts and fish is associated with a reduced risk of malnutrition or obesity, or other health problems [42]. Unfortunately, several poor dietary habits have been observed in pregnant women, including inadequate intake of fish, vegetables, milk and fermented milk drinks, alongside excessive consumption of sweets [44]. The most common dietary risk factors also include frequent consumption of processed foods (59.5%), insufficient exposure to the sun (52%) and insufficient consumption of fruits, fish and vegetables (43.5%). Consumption of ultra-processed foods with a lack of high-quality products such as fresh fruit, dairy, fish and seafood indicates a poor quality maternal diet, and according to a recent review, these behaviors are linked to negative perinatal outcomes, diabetes and preeclampsia [45] and fetal heart defects [46].

Numerous studies confirm that vitamin D and LC-PUFA are essential for pregnant women and the developing fetus because the body uses them for building and different metabolic processes [18,21,24,25,35]. However, there are no studies on the effect of vitamin D and long-chain fatty acid consumption and supplementation on the development of CHD in the fetus. In the context of public health, prevention of developmental disorders and prevention of congenital heart defects in the fetus are extremely important issues. Therefore, it is crucial to support women and men in the field of reproductive health through health education on rational and responsible offspring planning, taking into account a rational diet. Based on previous reports and our studies, it would be particularly important to assess the dietary intake of essential nutrients (vitamin D, LC-PUFA, folic acid) in the preconception period, together with the assessment of their concentration in the woman’s body. In this regard, education of parents-to-be with the support of a dietician in a preconception clinic should be considered. Training and courses for doctors, dieticians and nurses/midwives regarding the latest nutritional recommendations for pregnant women and women preparing to have a child would also be important. Materials including the latest nutritional and supplementation data and recommendations as well as developed leaflets for medical personnel and parents-to-be would also be helpful.

## 5. Strengths and Limitations of the Study

This study has several strengths. It provides detailed data on vitamin D and DHA supplementation (before pregnancy, at the start, and after the eighth week of pregnancy), as well as dietary intake and analysis of these nutrients, allowing for comprehensive control of these variables. The combined intake and supplementation in the context of CHD was also assessed. The maternal diet during pregnancy is relatively stable, with the period from the third to the eighth week of pregnancy being particularly critical for fetal cardiovascular development [2]. An additional advantage of this study was a simultaneous collection of clinical and nutritional history, along with accurate ultrasound diagnosis, in both the study and control groups, enabling direct comparison, as demonstrated in the previous study [16]. To our knowledge, there are still few studies examining both the intake and supplementation of vitamin D and LC-PUFA (including DHA] during the periconceptional period and pregnancy in relation to CHD. Therefore, our study fills this gap with the studies assessing the intake of these important nutrients.

The study’s limitations include the small sample size (particularly in the study group), which prevented a separate analysis of the associations between intake and supplementation and other subtypes of CHD, potentially revealing etiological differences [46]. In future studies, it would be advisable to expand the study group along with the control group—this should undoubtedly be taken into account. Additionally, we considered the possibility of recall bias and the underreporting of portion sizes. After the respondents had completed the interview questionnaire, a trained interviewer asked about food products, frequency and size of portions served, however, the patients may not have remembered exactly what, in what quantity and when they consumed the products.

Despite this, the FFQ questionnaire remains the most commonly used tool in surveys [47,48]. Recall bias regarding supplementation was also took into account. Finally, in addition to DHA, vitamin D, and folic acid (previously assessed), it should be mentioned that many other dietary factors/nutrients may affect the risk of developing CHD in the fetus, such as iron, magnesium, iodine, which have not been assessed so far and which may have some interactions [12]. Therefore, future studies should focus on assessing the combined effect of various dietary factors and other confounding factors, including age, education, childbirth, and others. Our next study will focus on such an assessment and analysis.

As indicated by other researchers, to enhance the reliability of the data, further studies could incorporate objective verification methods, such as assessing serum vitamin D concentration [11,15] and assessing (e.g., DHA level in blood) [12] or measuring fatty acids in phospholipids of erythrocyte membranes [35].

Our study may help identify risk and protective factors for congenital heart defects in the fetus in women planning a pregnancy and/or at the beginning of pregnancy with low intake of vitamin D or omega-3 fatty acids. However, further studies are needed on a larger study and control group.

## 6. Conclusions

There is insufficient evidence to state with certainty that a deficiency of vitamin D and LC-PUFA acids (including DHA) in the diet of women increases the risk of heart defects in the fetus.

Undoubtedly, the mother’s diet plays an important role in the proper course of pregnancy and affects the developing fetus by protecting against congenital heart defects. Vitamin D and omega-3 acids in the diet of a pregnant woman with additional supplementation may prove to be important elements protecting against heart defects, especially since diets at this time are very often deficient in these components and therefore supplementation may prove to be important, however, further studies in this area are needed.

Nutritional recommendations should include education for women preparing for pregnancy as well as healthcare workers, including doctors, dietitians, nurses, on the importance of increasing the intake of vitamin D, LC-PUFA acids in the preconception period and during pregnancy, which can be achieved by combining a rational diet containing the recommended intake of fish and supplements.

## Figures and Tables

**Table 1:** Association between the level of norm implementation for vitamin D, including the time of supplementation beginning, and the occurrence of congenital heart defects in the fetus.

Variables	CHD (A) (n = 79)		NHA (B) (n = 121)		
	N	%	N	%	OR (95%CI)
Vitamin D—diet					
Implementation below the norm	78	98.73	120	99.17	
Implementation normal	1	1.27	0	0	Ref.
Implementation above the norm	0	0	1	0.83	
	chi^2^ = 2.185; *p* = 0.335				
Vitamin D—implementation of the norm before pregnancy = diet + supplementation					
Supplementation before pregnancy and implementation of the norm	14	17.7	24	19.8	Ref.
No supplementation before pregnancy and not meeting the norm	65	82.3	97	80.2	1.15 (0.55–2.39)
chi^2^ = 0.139; *p* = 0.709					
Vitamin D-supplementation					
Supplementation before pregnancy	14	18.42	24	20.69	Ref.
Supplementation from 1 to 8 weeks of pregnancy	34	44.74	58	50.00	1.00 (0.41–2.47)
Supplementation above the 8th week of pregnancy	9	11.84	19	16.38	0.81 (0.29–2.39)
No supplementation	19	25.00	15	12.93	2.17 (0.80–5.90)
	chi^2^ = 5.469; *p* = 0.141				

CHD—congenital heart defects, NHA—normal heart anatomy.

**Table 2:** Association between the level of norm implementation for LC-PUFA and DHA supplementation, including the time of supplementation beginning, and the occurrence of congenital heart defects in the fetus.

Variables	CHD (A) (n = 79)		NHA (B) (n = 121)		
	N	%	N	%	OR (95%CI)
LC-PUFA—diet					
Implementation below the norm	39	49.37	51	42.15	0.87 (0.38–1.96)
Implementation normal	15	18.99	17	14.05	Ref.
Implementation above the norm	25	31.65	53	43.80	0.53 (0.23–1.25)
	chi^2^ = 3.093; *p* = 0.213				
LC-PUFA—implementation of the norm before pregnancy = diet + supplementation					
Supplementation before pregnancy and implementation of the norm	3	3.8	5	4.1	Ref.
No supplementation before pregnancy and not meeting the norm	76	96.2	116	95.9	0.91 (0.21–3.92)
chi^2^ = 0.014; *p* = 0.906					
DHA-supplementation					
Supplementation before pregnancy	5	6.49	5	4.24	Ref.
Supplementation from 1 to 8 weeks of pregnancy	23	29.87	34	28.81	0.68 (0.17–2.63)
Supplementation above the 8th week of pregnancy	21	27.27	41	34.75	0.51 (0.13–1.99)
No supplementation	28	36.36	38	32.20	0.74 (0.19–2.81)
	chi^2^ = 2.276; *p* = 0.517				

CHD—congenital heart defects, DHA—Docosahexaenoic Acid, LC-PUFA—Long Chain Polyunsaturated Fatty Acids, NHA—normal heart anatomy.

## Data Availability

The data presented in this paper are available upon request from the corresponding author. The data are not publicly available due to privacy and ethical restrictions.

## References

[1] Liu Y., Chen S., Zühlke L., Black G.C., Choy M.-K., Li N., Keavney B.D. (2019). Global birth prevalence of congenital heart defects 1970–2017: Updated systematic review and meta-analysis of 260 studies. Int. J. Epidemiol..

[2] Mires S., Caputo M., Overton T., Skerritt C. (2022). Maternal micronutrient deficiency and congenital heart disease risk: A systematic review of observational studies. Birth Defects Res..

[3] Walker K.C., Thorsteinsdottir F., Christesen H.T., Hjortdal V.E., Heitmann B.L., Specht I.O., Händel M.N. (2023). Vitamin D Supplementation and Vitamin D Status during Pregnancy and the Risk of Congenital Anomalies—A Systematic Review and Meta-Analysis. Nutrients.

[4] Amza M., Hamoud B.H., Sima R.-M., Dinu M.-D., Gorecki G.-P., Popescu M., Gică N., Poenaru M.-O., Pleș L. (2024). Docosahexaenoic Acid (DHA) and Eicosapentaenoic Acid (EPA)—Should They Be Mandatory Supplements in Pregnancy?. Biomedicines.

[5] Liu W., Ren L., Fang F., Chen R. (2024). Maternal pre-pregnancy overweight or obesity and risk of birth defects in offspring: Population-based cohort study. Acta Obstet. Gynecol. Scand..

[6] Adams B.J., Kirby K.J., Sorensen C.J., Pollard L.E., Audhya T. (2022). Evidence based recommendations for an optimal prenatal supplement for women in the US: Vitamins and related nutrients. Matern. Health Neonatol. Perinatol..

[7] Wendołowicz A., Stefańska E., Ostrowska L. (2014). Żywienie kobiet w okresie ciąży [Nutrition for Pregnant Women]. Med. Ogólna Nauk. Zdrowiu.

[8] Grzelak T., Suliga K., Sperling M., Pelczyńska M., Czyżewska K. (2016). Ocena stosowania suplementów diety wśród kobiet ciężarnych lub planujących ciążę [Assessment of the use of dietary supplements among pregnant women or those planning a pregnancy]. Forum Zaburzeń Metab..

[9] Aparicio E., Martín-Grau C., Hernández-Martinez C., Voltas N., Canals J., Arija V. (2021). Changes in fatty acid levels (saturated, monounsaturated and polyunsaturated) during pregnancy. BMC Pregnancy Childbirth.

[10] Bałtuć K. (2019). The influence of the vitamin d during pregnancy and in maternal and child health. Pol. Przegląd Nauk. Zdrowiu.

[11] Mokhtar A.W., Fawzy A., Allam M.R., Amer M.R., Hamed S.M. (2019). Maternal vitamin D level and vitamin D receptor gene polymorphism as a risk factor for congenital heart diseases in offspring; An Egyptian case-control study. Genes Dis..

[12] Gualtieri P., Frank G., Cianci R., Dominici F., Mappa I., Rizzo G., De Santis G.L., Bigioni G., Di Renzo L. (2024). Fish Consumption and DHA Supplementation during Pregnancy: Study of Gestational and Neonatal Outcomes. Nutrients.

[13] Derbyshire E.J., Birch C.S., Bonwick G.A., English A., Metcalfe P., Li W. (2024). Optimal omegas—Barriers and novel methods to narrow omega-3 gaps. A narrative review. Front. Nutr..

[14] Sley E.G., Rosen E.M., van ‘t Erve T.J., Sathyanarayana S., Barrett E.S., Nguyen R.H., Bush N.R., Milne G.L., Swan S.H., Ferguson K.K. (2020). Omega-3 fatty acid supplement use and oxidative stress levels in pregnancy. PLoS ONE.

[15] Koster M.P.H., van Duijn L., Krul-Poel Y., Laven J., Helbing W., Simsek S., Steegers-Theunissen R. (2018). A Compromised Maternal Vitamin D Status is Associated With Congenital Heart Defects in Offspring. Early Hum. Dev..

[16] Kolmaga A., Trafalska E., Gaszyńska E., Murlewska J., Witkowski S., Sylwestrzak O., Sokołowski Ł., Respondek-Liberska M., Strzelecka I. (2024). Folic Acid and Selected Risk Factors for Fetal Heart Defects—Preliminary Study Results. Nutrients.

[17] Wajszczyk B., Chwojnowska Z., Nasiadko D., Rybaczuk M. (2021). Instrukcja Korzystania Z Programu Dieta 6.0 Do Planowania I Bieżącej Oceny Żywienia Indywidualnego.

[18] Stoś K., Głowala A., Ziółkowska I., Jarosz M., Rychlik E., Stoś K., Charzewska J. (2020). Normy a suplementacja [Norms and supplementation]. Normy Żywienia Dla Populacji Polski I Ich Zastosowanie [Nutrition Standards for the Polish Population and Their Use].

[19] Stephenson J., Heslehurst N., Hall J., Schoenaker D.A., Hutchinson J., Cade J.E., Poston L., Barrett G., Crozier S.R., Barker M. (2018). Before the beginning: Nutrition and lifestyle in the preconception period and its importance for future health. Lancet.

[20] Cuartas S., Torre M. (2021). Metabolism and importance of polyunsaturated fatty acids in pregnancy and lactation. Rev. Cuba. Pediatría.

[21] Zimmer M., Sieroszewski P., Oszukowski P., Huras H., Fuchs T., Pawlosek A. (2020). Polish Society of Gynecologists and Obstetricians recommendations on supplementation in pregnancy. Ginekol. Pol..

[22] Çobanoğullari H., Ergoren M.C., Dundar M., Bertelli M., Tulay P. (2022). Periconceptional Mediterranean diet during pregnancy on children’s health. J. Prev. Med. Hyg..

[23] Palacios C., Kostiuk L.K., Peña-Rosas J.P. (2019). Vitamin D supplementation for women during pregnancy. Cochrane Database Syst. Rev..

[24] Sosnowska C. (2023). Wytyczne dotyczące profilaktyki i leczenia niedoboru witaminy D—Aktualizacja z 2023 r. w Polsce [Guidelines for Preventing and Treating Vitamin D Deficiency—Update as of 2023 in Poland]. Stand. Med. Pediatr..

[25] Koletzko B., Godfrey K.M., Poston L., Szajewska H., Van Goudoever J.B., De Waard M., Brands B., Grivell R.M., Deussen A.R., Dodd J.M. (2019). Nutrition during pregnancy, lactation, and early childhood and its implications for maternal and long-term child health: The EarlyNutrition Project recommendations. Ann. Nutr. Metab..

[26] Wierzejska R. (2018). Oszacowanie spożycia witaminy D i jej źródła w diecie kobiet ciężarnych [Estimation of vitamin D intake and its sources in the diet of pregnant women]. Zyw. Człowieka Metab..

[27] Agustina R., Rianda D., Lasepa W., Birahmatika F.S., Stajic V., Mufida R. (2023). Nutrient intakes of pregnant and lactating women in Indonesia and Malaysia: Systematic review and meta-analysis. Front. Nutr..

[28] Kocyłowski R., Lewicka I., Grzesiak M., Gaj Z., Sobańska A., Poznaniak J., von Kaisenberg C., Suliburska J. (2018). Assessment of dietary intake and mineral status in pregnant women. Arch. Gynecol. Obstet..

[29] Bärebring L., Amberntsson A., Winkvist A., Augustin H. (2018). Validation of Dietary Vitamin D Intake from Two Food Frequency Questionnaires, Using Food Records and the Biomarker 25-Hydroxyvitamin D among Pregnant Women. Nutrients.

[30] Jun S., Gahche J.J., Potischman N., Dwyer J.T.D., Guenther P.M.P., Sauder K.A., Bailey R.L.P. (2020). Dietary supplement use and its micronutrient contribution during pregnancy and lactation in the United States Shinyoung. Obstet. Gynecol..

[31] Dilli D., Doğan N.N., Örün U.A., Koç M., Zenciroğlu A., Karademir S., Akduman H. (2018). Maternal and neonatal micronutrient levels in newborns with CHD. Cardiol. Young.

[32] Jiang Y., Chen Y., Wei L., Zhang H., Zhang J., Zhou X., Zhu S., Du Y., Su R., Fang C. (2023). DHA supplementation and pregnancy complications. J. Transl. Med..

[33] Nordgren T.M., Lyden E., Anderson-Berry A., Hanson C. (2017). Omega-3 Fatty Acid Intake of Pregnant Women and Women of Childbearing Age in the United States: Potential for Deficiency?. Nutrients.

[34] Grot M., Grabowska K., Białek-Dratwa A., Grajek M. (2022). Pregnant women’s diets and fatty acid intake—A study of the frequency of intake of products that are sources of fatty acids among women in the third trimester of pregnancy. J. Educ. Health Sport.

[35] Nikolajeva K., Aizbalte O., Rezgale R., Cauce V., Zacs D., Meija L. (2024). The Intake of Omega-3 Fatty Acids, the Omega-3 Index in Pregnant Women, and Their Correlations with Gestational Length and Newborn Birth Weight. Nutrients.

[36] Masina M., Medithi S., Muley A. (2023). Impact of maternal essential fatty acid intake on the birth weight of infants. J. Mother Child.

[37] Wiesner A., Paśko P. (2021). Stosowanie suplementów u kobiet ciężarnych w świetle najnowszych rekomendacji Polskiego Towarzystwa Ginekologów i Położników [The use of supplements in pregnant women in the light of the latest recommendations of the Polish Society of Gynecologists and Obstetricians]. Farm Pol..

[38] Roke K., Rattner J., Brauer P., Mutch D.M. (2018). Awareness of Omega-3 fatty acids and possible health effects among young adults. Can. J. Diet. Pract. Res..

[39] Wierzejska R., Jarosz M., Wojda B., Siuba-Strzelińska M. (2018). Dietetyczne spożycie DHA w czasie ciąży: Znaczna różnica między rzeczywistym spożyciem a aktualnymi zaleceniami żywieniowymi [Dietary intake of DHA during pregnancy: Significant difference between actual intake and current dietary recommendations]. Rocz. Panstw. Zakl. Hig..

[40] Capra L., Tezza G., Mazzei F., Boner A.L. (2013). The origins of health and disease: The influence of maternal diseases and lifestyle during. Ital. J. Pediatr..

[41] Obermann-Borst S.A., Vujkovic M., de Vries J.H., Wildhagen M.F., Looman C.W., de Jonge R., Steegers E.A.P., Steegers-Theunissen R.P.M. (2011). A maternal dietary pattern characterised by fish and seafood in association with the risk of congenital heart defects in the offspring. BJOG.

[42] Gallo L.A., Steane S.E., Young S.L., de Jersey S., Schoenaker D.A.J.M., Borg D.J., Lockett J., Collins C.E., Perkins A.V., Kumar S. (2024). Dietary supplements, guideline alignment and biochemical nutrient status in pregnancy: Findings from the Queensland Family Cohort pilot study. Matern. Child Nutr..

[43] Lowensohn R.I., Stadler D.D., Naze C. (2016). Current Concepts of Maternal Nutrition. Obstet. Gynecol. Surv..

[44] Ługowska K., Kolanowski W. (2019). The Nutritional Behaviour of Pregnant Women in Poland. Int. J. Environ. Res. Public Health.

[45] de Oliveira P.G., de Sousa J.M., Assunção D.G.F., de Araujo E.K.S., Bezerra D.S., Dametto J.F.d.S., Ribeiro K.D.d.S. (2022). Impacts of Consumption of Ultra-Processed Foods on the Maternal-Child Health: A Systematic Review. Front. Nutr..

[46] Yang J., Cheng Y., Zeng L., Dang S., Yan H. (2021). Maternal Diet Diversity During Pregnancy and Congenital Heart Defects: A Case-Control Study. Eur. J. Clin. Nutr..

[47] Zakaria H., Abusanana S., Mussa B.M., Al Dhaheri A.S., Stojanovska L., Mohamad M.N., Saleh S.T., Ali H.I., Ismail L.C. (2023). The Role of Lifestyle Interventions in the Prevention and Treatment of Gestational Diabetes Mellitus. Medicina.

[48] Cheng Z., Gu R., Lian Z., Gu H.F. (2022). Evaluation of the association between maternal folic acid supplementation and the risk of congenital heart disease: A systematic review and meta-analysis. Nutr. J..

